# CD19+ lineage chimerism, an early biomarker after anti-CD19 CAR-T cell therapy in patients previously receiving a hematopoietic stem cell transplantation

**DOI:** 10.3389/fimmu.2022.960412

**Published:** 2022-08-08

**Authors:** Isabel Martínez-Romera, Víctor Galán-Gómez, Berta González-Martínez, Pilar Guerra García, Sonsoles San Román Pacheco, Dolores Corral Sánchez, Yasmina Mozo del Castillo, David Bueno Sánchez, Luisa Sisinni, Alba González Guerrero, Serafin Castellano Dámaso, Elena Sánchez Zapardiel, Beatriz Ruz Caracuel, Antonio Balas Pérez, Antonio Pérez-Martínez

**Affiliations:** ^1^ Pediatric Hematology and Oncology Department, La Paz University Hospital, Madrid, Spain; ^2^ Immunology Department, La Paz University Hospital, Madrid, Spain; ^3^ Institute of Medical and Molecular Genetics (INGEMM), La Paz University Hospital, Madrid, Spain; ^4^ Center of Blood Transfusion, Madrid, Spain; ^5^ La Paz University Hospital Research Institute (IdiPAZ), Madrid, Spain; ^6^ Pediatric Department, Universidad Autónoma, Madrid, Spain

**Keywords:** pediatric acute lymphoblastic leukemia (ALL), CAR-T cell therapy, hematopoietic stem cell (HSC) transplantation, lineage chimerism, biomarker

## Abstract

Treatment targeting CD19 by a chimeric antigen receptor expressed on T cells (anti-CD19 CAR-T) has led to a breakthrough in the management and treatment of relapsed and refractory B- cell acute lymphoblastic leukemia (B-ALL). After infusion, the efficacy of anti-CD19 CAR-T is monitored by bone marrow negative minimal residual disease and the absence of peripheral CD19^+^ B lymphocytes (B-cell aplasia). In patients who have received an allogenic Hematopoietic Stem Cell Transplantation (HSCT) prior to treatment with anti-CD19 CAR-T, monitoring lineage-specific chimerism could be helpful. We found that on 4 patients who received anti-CD19 CAR-T cells after HSCT and achieved early complete response, CD19^+^ lineage mixed chimerism but not CD3^+^ lineage mixed chimerism monitored by molecular techniques anticipated earlier than B-cell aplasia determined by flow cytometry, lack of effectiveness of anti-CD19 CAR-T and leukemia relapse. Donor lymphocyte infusions (DLIs) did not prevent relapse but recovered CD3^+^ full donor chimerism. We suggest that continuous lineage chimerism analysis should be done routinely in patients who receive anti-CD19 CAR-T cells after HSCT and achieve complete remission because it can support early treatment intervention. However, the role of DLI in this setting is unclear, so further prospective studies should be developed.

## Introduction

Leukemia relapse remains the main treatment failure after chimeric antigenic receptor targeting CD19 expressed on T lymphocytes (antiCD19 CAR-T). Prolonged B-cell aplasia is a well-known side effect after anti-CD19 CAR-T due to on-target, off-tumor effect (1). Early B-cell recovery, normally 6 months after infusion, determined by flow cytometry, indicates anti-CD19 CAR-T loss of function, limited expansion and persistence. It is used as a biomarker to recommend allogenic hematopoietic stem cell transplantation (HSCT) or anti-CD19 CAR-T reinfusion.

HSCT is considered standard treatment for high-risk B-cell leukemia, requiring chimerism monitoring with short tandem repeats (STR) by polymerase chain reaction (PCR) to test graft function and predict patient outcome (2). Prospective studies show that patients with B-cell acute leukemia with increasing mixed chimerism have greater risk of relapse (3, 4), thus immunosuppression withdrawal and/or administration of donor lymphocyte infusion (DLI) (5) is recommended.

Clinical data report a wide range (20-81%) of patients who received anti-CD19 CAR-T after at least one previous HSCT (6). However, in these patients, chimerism data of the anti-CD19 CAR-T cell product before infusion are scarce and chimerism monitoring after infusion is not well established. The source of chimerism looks not relevant because several clinical trials show no difference in the complete remission rate whether patients received previous HSCT or not (7, 8). However, we would expect full donor chimerism in patients who achieved early complete remission and the appearance of recipient cells would be indicative of early stage leukemia relapse and could require treatment (9–11) with DLIs, anti-CD19 CAR-T reinfusion or a second HSCT. Hence, post remission chimerism monitoring could be helpful.

## Methods

Chimerism analysis was performed by PCR-STR using nine polymorphic, autosomal non-coding STR loci, and Amelogenin. Oligonucleotide amplification mixes were designed and validated in-house. Specificity, informativeness and sensitivity were established by using local artificial DNA mixtures and external proficiency testing. Amplification conditions were defined to achieve the detection of at least a 2% of the minor component. In order to avoid possible variations in the sensitivity, at least two informative STR systems were employed to give a final result.

The CD3^+^ and CD19^+^ lymphocyte subsets were obtained by using direct immunomagnetic procedures (Stemcell Technologies, Cambridge, MA). The purity of the isolated lymphocytes samples obtained with these reagents was previously validated by flow cytometry, and a minimum of 95% of the target lymphocyte subset was consistently obtained.

Amplifications used 2 ng of genomic DNA with subsequent capillary electrophoresis in a Genetic Analyzer 3130xl (Applied Biosystems; Waltham, MA) according to manufacturer recommendations, with Fragment Analysis 4.0 software (Applied Biosystems).

The HIV viral load was measured by quantitative real time PCR (COBAS AmpliPrep/COBAS TaqMan HIV Test by Roche Molecular diagnostics). During CAR-T engineering, lentiviral vectors which contain portions of the HIV viral genome are used to transduce T-cells of patient’s *ex-vivo*, becoming integrated into the genomes of these transfected cells that later are infused into patients. The research protocol for generating tisagenlecleucel use a lentiviral vector that contained the HIV LTR and gag gene sequences and the COBAS AmpliPrep/COBAS TaqMan HIV Test detects these both sequences, so is accepted to use it as an indirect method to measure the CAR-T.

Immunophenotyping of peripheral blood B lymphocytes (CD19+) was performed by a multiparametric flow cytometry analysis during the follow-up. Peripheral blood was stained for surface markers with the following fluorochrome-conjugated anti-CD19 antibody (Beckman Coulter): anti-CD19 PC7 (clone J3-119). Cells were acquired on DxFlex cytometer (Beckman Coulter). The analysis was performed with Kaluza™ Analysis Software v2.1 (Beckman Coulter).

## Result and discussion

### Patients and results

Clinical and biological features of each patient and frontline treatment regimen are shown in **Table 1**. All patients received fludarabine and cyclophosphamide as lymphodepletion regimen.

**Table 1:** Summarize of patients with diagnosis, frontline treatment, salvage treatment, outcome and follow up.

**Patient**	**1**	**2**	**3**	**4**	
	**Patient 1**	**Patient 2**	**Patient 3**	**Patient 4**	
**Gender**	M	F	M	M	
**Age at diagnosis (years)**	17	0.5	4	2	
**Diagnosis**	B-ALL	B-ALL	B-ALL	B-ALL	
**Prior treatment regimen**	SEHOP-PHETEMA 2013	INTERFANT06	SEHOP-PHETEMA 2013	SEHOP-PHETEMA 2013	
**1^st^ Allo-HSCT (before CAR-T cell therapy)**	MUD	MUD	MRD	MUD	
**Number of CAR-T cells/Kg**	1.7 x 10^6^	2.7 x 10^6^	3.3 x 10^6^	2.3 x 10^6^	
**Chimerism during follow up**					
** Bone marrow mononuclear cells**	Complete	Complete	Complete	Complete	
** Maximum autologous % CD3^+^ cells (months after CAR-T cell therapy) in PB**	Mixed 7%. (4.5)	Complete	Complete	Complete	
** Maximum autologous % CD19^+^ cells (months after CAR-T cell therapy) in PB**	Complete	Mixed 5% (1.5)	Mixed 6% (3)	Mixed 7% (6.5)	
**B cell aplasia (months after CAR-T cell therapy)**	Maintained	Maintained	Lost (3)	Maintained	
**Relapse type (months after CAR-T cell therapy)**	No	Yes, node CD19^-^ (9)	No	Yes, medullar CD19^-^ (6)	
**Donor lymphocytes infusion (number)**	7	8	0	3	
**2^nd^ Allo-HSCT after CAR-T cell therapy**	No	Yes, haplo	Yes, haplo	Yes, haplo	
**Current status (months after 2^nd^ HSCT)**	Alive in CR	Alive in CR (5)	Alive in CR (12)	Alive in CR (3)	

F, female; M, male; Allo-HSCT, Allogeneic hematopoietic stem cell transplant; CAR-T, chimeric antigenic receptor T cell; MUD, matched unrelated donor; MRD, matched related donor;PB, peripheral blood; CR, Complete remission.

#### Patient 1

**Figure 1:**
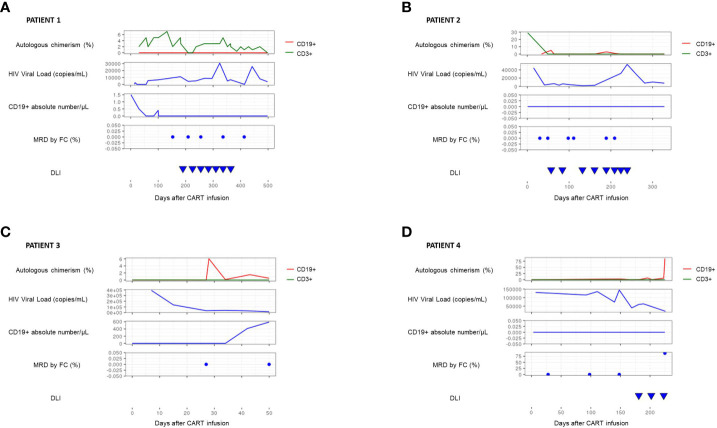
This graph summarizes the evolution, in each patient, of several biomarkers in the post-CART period. These are the autologous chimerism in CD3 lineage (green line) and CD19 lineage (red line), the HIV viral load measured by PCR, the absolute number of CD19 lymphocytes and the minimal residual disease, measured both by flow cytometry. In patients #1 **(A)** and #2 **(B)**, it is observed that the presence of chimerism in the CD3 lineage is not associated with a loss of CART function or with relapse and can also be reversed with periodic infusions of donor lymphocytes. In patient #3 **(C)**, it is shown that the appearance of autologous CD19 chimerism precede the loss of B lymphocyte aplasia and decreased HIV load, constituting an earlier marker of risk of disease relapse. Finally, in patient #4 **(D)**, it is evident that the mixed chimera in CD19 lineage is directly associated with the relapse of the disease, being in this case much more useful than monitoring the B lymphocyte aplasia, which remain unchanged in this patient.

A 17-year-old male diagnosed with high-risk t (1:19) B-ALL relapsing 20 months after a matched unrelated donor (MUD) HSCT, received tisagenlecleucel, 1.7 x 10^6^ cells/kg. Developed grade II cytokine release syndrome (CRS). Early evaluation showed complete response and full donor chimerism. Bone marrow and peripheral blood chimerism, minimal residual disease (MRD), peripheral HIV viral load and B-cell aplasia were monitored. He presented peripheral blood mixed chimerism in CD3^+^, with a gradual maximal 7% increase 4.5 months after anti-CD19 CAR-T infusion. HIV viral load ranged from a minimum, 2 weeks after infusion, of 4.82 x 10^1^cop/ml to a maximum of 3.07 x 10^4^ cop/ml, eleven months post infusion. B-cell aplasia and negative MRD maintained. Infused donor CD45RA^-^ T cells monthly to restore T cell donor chimerism and minimize graft versus host disease (GvHD). Mixed chimerism decreased progressively reaching complete donor chimerism 8 months later, after 7 DLIs. Currently, 24 months after anti-CD19 CAR-T infusion, he is in complete remission (CR) and GvHD free (**Figure 1A**).

#### Patient 2

A 13-month female with mixed-lineage leukemia (*MLL*)-*rearranged* leukemia B-ALL relapsing 5 months after MUD, received tisagenlecleucel 2.7 x 10^6^ cells/kg. Presented grade II CRS. Early evaluation showed CR and full donor chimerism. Soon after (1.5 months), she presented recipient (5%) CD19^+^ chimerism but negative MRD, maintaining B-cell aplasia and detecting HIV viral load (range of 3.3 x 10^3^ cop/ml to 3.32 x 10^4^ cop/ml throughout follow-up). Infused monthly CD45RA^-^ T cells achieving full donor chimerism after 8 DLIs. However, 9 months after antiCD19 CAR-T infusion, she presented an isolated extramedullary relapse (cervical node relapse). She underwent resection and a second HSCT (haploidentical donor). Currently, she continues leukemia-free 5 months after HSCT, with full donor chimerism (**Figure 1B**).

#### Patient 3

A 5-year-old male with a CRLF2 overexpression B-ALL relapsing nineteen months after a match related donor (MRD), received tisagenlecleucel 3.28 x 10^6^ cells/kg. After infusion, no CRS. First evaluation after CAR-T therapy, found CD19^+^ recipient chimerism, maximal 6%, and presence of CD19^+^ B lymphocytes (maximal 590/μL), with a progressive decrease of HIV viral load (from 3.9 x 10^5^ cop/ml to 1.55 x 10^4^ cop/ml) but negative MRD. Four months after anti-CD19 CAR-T infusion, second HSCT (haploidentical donor). Currently, he continues leukemia-free 12 months after haplo-HSCT with full donor chimerism (**Figure 1C**).

#### Patient 4

A 3-year-old male with RUNX1+ B-ALL relapsing nine months after MUD HSCT, received tisagenlecleucel 2.3 x 10^6^ cell/kg. Developed grade II CRS and grade III immune effector cell-associated neurotoxicity syndrome *(ICANS)* requiring treatment with tocilizumab and steroids, with good response and recovery. Early evaluation showed complete response and full donor chimerism. He presented peripheral blood mixed chimerism in CD19^+^ cells at 5 months of anti-CD19 CAR-T infusion. HIV viral load decreased progressively (from 4.9 x 10^5^ cop/ml to 1.98 x 10^4^ cop/ml) but B-cell aplasia and negative MRD maintained. On day +180 after infusion, monthly donor CD45RA^-^ T cell infusions, to restore T cell donor chimerism and minimize GvHD. After the third DLI, recipient CD19^+^ chimerism persisted and an isolated medullary relapse was confirmed. Patient received chemotherapy and haploidentical HSCT. Relapse 5 months later and, after salvage therapy, a third haploidentical HSCT was performed. Currently in CR 3 months after transplant (**Figure 1D**).

### Discussion

Including lineage specific chimerism in follow-up of patients with r/r B-ALL receiving anti-CD19^+^ CAR-T therapy after an allogeneic HSCT, has proven to be very useful. We believe PCR-STR for CD19^+^ and/or CD3^+^ lineage-specific analysis is the best method because it requires a very low amount of DNA.

In candidates for CAR-T cell therapy after a previous HSCT, among the CD3^+^ cells, mixed chimerism with a maximum of 5% autologous cells before apheresis is accepted (12). This may remain after CAR-T infusion and is allowed because, as shown in patient #1, it is not related with a worse outcome, and can be reverted with serial DLIs.

Nevertheless, our findings (patients #2 and #4) show the loss of completed chimerism observed in the CD19^+^ cells subset could be associated with a higher risk of relapse, even if not accompanied by a loss of CD19^+^ lymphocyte aplasia or a significant decrease in HIV viral load (patient #2), being the last one an indirect biomarker which could associate cross-reactions with HIV infection, leading to false-positive results (13–15). Although there is not enough evidence on optimal treatment in this case, our findings, supported by other publications (16), suggest that DLI may be not as effective as in other settings (17). Given these are usually very heavily pre-treated patients, they have probably developed resistance to DLIs regardless of a return to complete B lineage chimerism, as in patient #2.

In patient #3, mixed chimerism of the CD19^+^ line is an earlier marker of relapse than the loss of B lymphocyte aplasia. T

A specific loss of chimerism of the B lineage suggests that the minimum number of CD19 B lymphocytes detected would represent a very early stage of leukemia relapse, since they imply not only the loss of the effect of the allogeneic graft infused in the HSCT but also a loss of CAR-T antileukemia activity, whose effect can only be guaranteed if B cell aplasia is maintained. Thus, monitoring lineage specific chimerism in the first 6-12 months after CAR-T therapy could be crucial to anticipate interventions before relapse, as occurs today with the loss of B lymphocyte aplasia.

## Data availability statement

The original contributions presented in the study are included in the article/supplementary material. Further inquiries can be directed to the corresponding author.

## Author contributions

Contribution: IM-R, BG-M, and VG-G fulfilled the clinical and biological database; AB performed the chimerism molecular study; IM-R and BR made the graphic design; ESZ performed the immunological study; IM-R, AP-M, and AB wrote the paper and all authors discussed the results and commented on the paper. All authors contributed to the article and approved the submitted version.

## Funding

This work was supported by the National Health Service of Spain, Carlos III Health Institute (ISCIII), FONDOS FEDER grant (FIS) PI18/01301 and ICI19/00052 and CRIS Foundation to Beat Cancer, https://criscancer.org/en/.

## Acknowledgments

University Hospital La Paz and CRIS Cancer Unit provided the clinical data. Molecular analysis is supported by Transfusion Centre of Madrid.

## Conflict of interest

The authors declare that the research was conducted in the absence of any commercial or financial relationships that could be construed as a potential conflict of interest.

## Publisher’s note

All claims expressed in this article are solely those of the authors and do not necessarily represent those of their affiliated organizations, or those of the publisher, the editors and the reviewers. Any product that may be evaluated in this article, or claim that may be made by its manufacturer, is not guaranteed or endorsed by the publisher.
